# Diiodido{4-nitro-2-[2-(piperidin-1-yl)ethyl­imino­meth­yl]phenolato}zinc(II)

**DOI:** 10.1107/S1600536809040495

**Published:** 2009-10-10

**Authors:** Xue-Wen Zhu, Xu-Zhao Yang, Chun-Xia Zhang, Gang-Sen Li, Zhi-Gang Yin

**Affiliations:** a Key Laboratory of Surface and Interface Science of Henan, School of Material & Chemical Engineering, Zhengzhou University of Light Industry, Zhengzhou 450002, People’s Republic of China

## Abstract

In the title complex, [ZnI_2_(C_14_H_19_N_3_O_3_)], the Zn^II^ atom is four-coordinated by the imine N and phenolate O atoms of the Schiff base ligand, and by two iodide ions in a distorted tetra­hedral coordination. In the crystal structure, mol­ecules are linked through inter­molecular N—H⋯O hydrogen bonds, forming dimers.

## Related literature

For background to the chemistry of Schiff base complexes, see: Ali *et al.* (2008 ▶); Biswas *et al.* (2008 ▶); Chen *et al.* (2008 ▶); Darensbourg & Frantz (2007 ▶); Habibi *et al.* (2007 ▶); Kawamoto *et al.* (2008 ▶); Lipscomb & Sträter (1996 ▶); Tomat *et al.* (2007 ▶); Wu *et al.* (2008 ▶); Yuan *et al.* (2007 ▶). For related structures see: Zhu (2008 ▶); Zhu & Yang (2008*a* ▶,*b* ▶,*c* ▶); Qiu (2006*a* ▶,*b* ▶); Wei *et al.* (2007 ▶); Zhu *et al.* (2007 ▶).
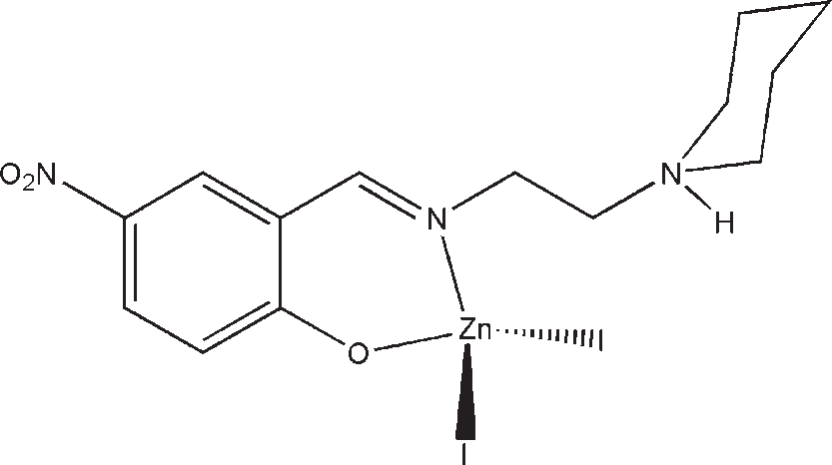


## Experimental

### 

#### Crystal data

[ZnI_2_(C_14_H_19_N_3_O_3_)]
*M* *_r_* = 596.49
Triclinic, 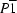

*a* = 8.7467 (2) Å
*b* = 10.7114 (3) Å
*c* = 10.9541 (2) Å
α = 89.553 (2)°
β = 89.334 (2)°
γ = 68.984 (2)°
*V* = 957.94 (4) Å^3^
*Z* = 2
Mo *K*α radiation
μ = 4.52 mm^−1^
*T* = 298 K
0.20 × 0.20 × 0.18 mm

#### Data collection

Bruker APEXII CCD area-detector diffractometer
Absorption correction: multi-scan (*SADABS*; Sheldrick, 2004 ▶) *T*_min_ = 0.465, *T*_max_ = 0.497
5795 measured reflections
4034 independent reflections
2994 reflections with *I* > 2σ(*I*)
*R*_int_ = 0.017

#### Refinement

*R*[*F* ^2^ > 2σ(*F* ^2^)] = 0.036
*wR*(*F* ^2^) = 0.080
*S* = 1.01
4034 reflections
211 parameters
1 restraint
H atoms treated by a mixture of independent and constrained refinement
Δρ_max_ = 0.50 e Å^−3^
Δρ_min_ = −0.62 e Å^−3^

### 

Data collection: *APEX2* (Bruker, 2004 ▶); cell refinement: *SAINT* (Bruker, 2004 ▶); data reduction: *SAINT*; program(s) used to solve structure: *SHELXS97* (Sheldrick, 2008 ▶); program(s) used to refine structure: *SHELXL97* (Sheldrick, 2008 ▶); molecular graphics: *SHELXTL* (Sheldrick, 2008 ▶); software used to prepare material for publication: *SHELXL97*.

## Supplementary Material

Crystal structure: contains datablocks global, I. DOI: 10.1107/S1600536809040495/om2281sup1.cif

Structure factors: contains datablocks I. DOI: 10.1107/S1600536809040495/om2281Isup2.hkl

Additional supplementary materials:  crystallographic information; 3D view; checkCIF report

## Figures and Tables

**Table:** 

Zn1—O1	1.948 (3)
Zn1—N1	2.037 (3)
Zn1—I2	2.5666 (6)
Zn1—I1	2.5690 (6)

**Table:** 

O1—Zn1—N1	94.96 (13)
O1—Zn1—I2	107.27 (10)
N1—Zn1—I2	113.81 (10)
O1—Zn1—I1	121.56 (10)
N1—Zn1—I1	102.97 (10)
I2—Zn1—I1	114.61 (2)

**Table 2:** Hydrogen-bond geometry (Å, °)

*D*—H⋯*A*	*D*—H	H⋯*A*	*D*⋯*A*	*D*—H⋯*A*
N2—H2⋯O2^i^	0.89 (4)	2.00 (3)	2.777 (5)	144 (5)

Symmetry code: (i) 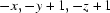.

**Fig. 1.**
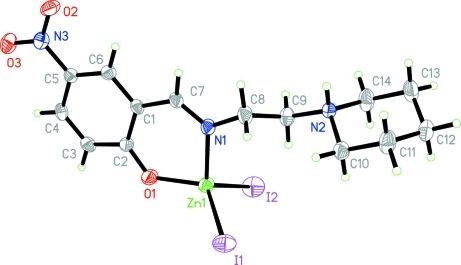
The molecular structure of the title complex, with ellipsoids drawn at the 30% probability level.

**Fig. 2.**
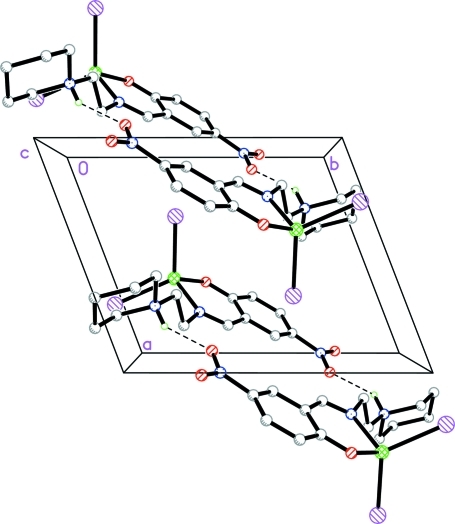
The crystal packing of the title complex, viewed along the c axis. Intermolecular hydrogen bonds are shown as dashed lines.

## supplementary crystallographic information

### Comment

Schiff bases are interesting ligands in coordination chemistry (Biswas *et al.*, 2008; Wu *et al.*, 2008; Kawamoto *et al.*, 2008; Ali *et al.*, 2008; Habibi *et al.*, 2007), and their complexes have been investigated in many fields (Chen *et al.*, 2008; Yuan *et al.*, 2007; Tomat *et al.*, 2007; Darensbourg & Frantz, 2007). Zinc(II) is an important element in biological systems and functions as the active site of hydrolytic enzymes, such as carboxypeptidase and carbonic anhydrase where it is in a hard-donor coordination environment of nitrogen and oxygen ligands (Lipscomb & Sträter, 1996). Recently, we have reported a few Schiff base zinc complexes (Zhu, 2008; Zhu & Yang, 2008a,b,c). In this paper, the title new zinc(II) complex, Fig. 1, is reported.

In the title complex, the Zn^II^ atom is four-coordinated by the imine N and phenolate O atoms of the Schiff base ligand, and by two iodide ions in a tetrahedral coordination. The coordinate bond lengths (Table 1) are typical and comparable to the corresponding values observed in the Schiff base zinc complexes we reported previously and other similar Schiff base zinc complexes (Zhu *et al.*, 2007; Wei *et al.*, 2007; Qiu, 2006*a*,b).

In the crystal structure, molecules are linked through intermolecular N—H···O hydrogen bonds, forming dimers (Table 2, Fig. 2).

### Experimental

The Schiff base compound was prepared by the condensation of equimolar amounts of 5-nitrosalicylaldehyde with 2-piperidin-1-ylethylamine in a methanol solution. The complex was prepared by the following method. To an anhydrous methanol solution (5 ml) of ZnI_2_ (31.9 mg, 0.1 mmol) was added a methanol solution (10 ml) of the Schiff base compound (27.7 mg, 0.1 mmol) with stirring. The mixture was stirred for 30 min at room temperature and filtered. Upon keeping the filtrate in air for a few days, colorless block-shaped crystals were formed.

### Refinement

H2 was located from a difference Fourier map and refined isotropically, with N—H distance restrained to 0.90 (1) Å. Other H atoms were placed in geometrically idealized positions and constrained to ride on their parent atoms, with C—H distances in the range 0.93–0.97 Å, and with *U*_iso_(H) = 1.2*U*_eq_(C).

### Crystal data

**Table:**

[ZnI_2_(C_14_H_19_N_3_O_3_)]	*Z* = 2
*M**_r_* = 596.49	*F*(000) = 568
Triclinic, *P*1	*D*_x_ = 2.068 Mg m^−^^3^
*a* = 8.7467 (2) Å	Mo *K*α radiation, λ = 0.71073 Å
*b* = 10.7114 (3) Å	Cell parameters from 1800 reflections
*c* = 10.9541 (2) Å	θ = 2.6–25.5°
α = 89.553 (2)°	µ = 4.52 mm^−^^1^
β = 89.334 (2)°	*T* = 298 K
γ = 68.984 (2)°	Block, colorless
*V* = 957.94 (4) Å^3^	0.20 × 0.20 × 0.18 mm

### Data collection

**Table:**

Bruker APEXII CCD area-detector diffractometer	4034 independent reflections
Radiation source: fine-focus sealed tube	2994 reflections with *I* > 2σ(*I*)
graphite	*R*_int_ = 0.017
ω scans	θ_max_ = 27.0°, θ_min_ = 1.9°
Absorption correction: multi-scan (*SADABS*; Sheldrick, 2004)	*h* = −11→10
*T*_min_ = 0.465, *T*_max_ = 0.497	*k* = −11→13
5795 measured reflections	*l* = −13→13

### Refinement

**Table:**

Refinement on *F*^2^	Primary atom site location: structure-invariant direct methods
Least-squares matrix: full	Secondary atom site location: difference Fourier map
*R*[*F*^2^ > 2σ(*F*^2^)] = 0.036	Hydrogen site location: inferred from neighbouring sites
*wR*(*F*^2^) = 0.080	H atoms treated by a mixture of independent and constrained refinement
*S* = 1.01	*w* = 1/[σ^2^(*F*_o_^2^) + (0.0311*P*)^2^ + 0.4408*P*] where *P* = (*F*_o_^2^ + 2*F*_c_^2^)/3
4034 reflections	(Δ/σ)_max_ = 0.001
211 parameters	Δρ_max_ = 0.50 e Å^−^^3^
1 restraint	Δρ_min_ = −0.62 e Å^−^^3^

### Special details

**Table:**

Geometry. All esds (except the esd in the dihedral angle between two l.s. planes) are estimated using the full covariance matrix. The cell esds are taken into account individually in the estimation of esds in distances, angles and torsion angles; correlations between esds in cell parameters are only used when they are defined by crystal symmetry. An approximate (isotropic) treatment of cell esds is used for estimating esds involving l.s. planes.
Refinement. Refinement of F^2^ against ALL reflections. The weighted R-factor wR and goodness of fit S are based on F^2^, conventional R-factors R are based on F, with F set to zero for negative F^2^. The threshold expression of F^2^ > 2sigma(F^2^) is used only for calculating R-factors(gt) etc. and is not relevant to the choice of reflections for refinement. R-factors based on F^2^ are statistically about twice as large as those based on F, and R- factors based on ALL data will be even larger.

### Fractional atomic coordinates and isotropic or equivalent isotropic displacement parameters (Å^2^)

**Table:**

	*x*	*y*	*z*	*U*_iso_*/*U*_eq_	
Zn1	0.38637 (6)	0.75022 (5)	0.70365 (5)	0.04264 (14)	
I1	0.26005 (4)	1.00680 (3)	0.70856 (3)	0.06259 (13)	
I2	0.68572 (4)	0.65555 (4)	0.63039 (4)	0.07424 (14)	
N1	0.2296 (4)	0.7001 (4)	0.5954 (3)	0.0390 (8)	
N2	0.2722 (4)	0.8331 (3)	0.2827 (3)	0.0363 (8)	
N3	0.0106 (4)	0.3085 (3)	0.8808 (3)	0.0435 (9)	
O1	0.3662 (4)	0.6417 (3)	0.8421 (3)	0.0533 (8)	
O2	−0.0766 (4)	0.3055 (3)	0.7938 (3)	0.0579 (9)	
O3	0.0124 (4)	0.2483 (3)	0.9762 (3)	0.0587 (9)	
C1	0.1913 (5)	0.5441 (4)	0.7469 (3)	0.0345 (9)	
C2	0.2852 (5)	0.5627 (4)	0.8453 (4)	0.0355 (9)	
C3	0.2900 (5)	0.4870 (4)	0.9535 (4)	0.0415 (10)	
H3	0.3534	0.4954	1.0183	0.050*	
C4	0.2054 (5)	0.4026 (4)	0.9657 (4)	0.0393 (10)	
H4	0.2104	0.3549	1.0377	0.047*	
C5	0.1107 (5)	0.3891 (4)	0.8682 (3)	0.0344 (9)	
C6	0.1057 (5)	0.4572 (4)	0.7614 (4)	0.0378 (9)	
H6	0.0438	0.4454	0.6970	0.045*	
C7	0.1648 (5)	0.6156 (4)	0.6312 (4)	0.0378 (10)	
H7	0.0934	0.5985	0.5774	0.045*	
C8	0.1724 (5)	0.7702 (5)	0.4791 (4)	0.0482 (11)	
H8A	0.1146	0.8647	0.4939	0.058*	
H8B	0.0975	0.7346	0.4405	0.058*	
C9	0.3181 (5)	0.7512 (5)	0.3965 (4)	0.0459 (11)	
H9A	0.3989	0.7758	0.4401	0.055*	
H9B	0.3676	0.6575	0.3748	0.055*	
C10	0.2279 (6)	0.9798 (4)	0.3042 (4)	0.0508 (12)	
H10A	0.3208	0.9959	0.3385	0.061*	
H10B	0.1380	1.0103	0.3626	0.061*	
C11	0.1785 (6)	1.0575 (5)	0.1866 (4)	0.0569 (13)	
H11A	0.1527	1.1519	0.2022	0.068*	
H11B	0.0809	1.0462	0.1553	0.068*	
C12	0.3150 (6)	1.0100 (5)	0.0919 (4)	0.0537 (12)	
H12A	0.2793	1.0582	0.0159	0.064*	
H12B	0.4100	1.0277	0.1200	0.064*	
C13	0.3597 (7)	0.8626 (5)	0.0713 (4)	0.0609 (14)	
H13A	0.4504	0.8317	0.0137	0.073*	
H13B	0.2672	0.8464	0.0362	0.073*	
C14	0.4070 (6)	0.7853 (5)	0.1887 (4)	0.0609 (14)	
H14A	0.4314	0.6911	0.1730	0.073*	
H14B	0.5052	0.7954	0.2201	0.073*	
H2	0.183 (4)	0.825 (5)	0.250 (4)	0.080*	

### Atomic displacement parameters (Å^2^)

**Table:**

	*U*^11^	*U*^22^	*U*^33^	*U*^12^	*U*^13^	*U*^23^
Zn1	0.0420 (3)	0.0466 (3)	0.0436 (3)	−0.0209 (2)	−0.0078 (2)	0.0040 (2)
I1	0.0622 (2)	0.0466 (2)	0.0738 (3)	−0.01273 (16)	−0.01062 (18)	−0.00726 (17)
I2	0.03816 (19)	0.0782 (3)	0.0974 (3)	−0.00982 (18)	−0.00365 (18)	−0.0088 (2)
N1	0.0397 (19)	0.047 (2)	0.0332 (19)	−0.0191 (17)	−0.0049 (16)	0.0070 (16)
N2	0.040 (2)	0.039 (2)	0.0326 (19)	−0.0174 (16)	−0.0002 (15)	0.0030 (15)
N3	0.042 (2)	0.037 (2)	0.048 (2)	−0.0101 (17)	−0.0009 (18)	0.0072 (17)
O1	0.065 (2)	0.070 (2)	0.0409 (17)	−0.0426 (19)	−0.0175 (15)	0.0099 (16)
O2	0.068 (2)	0.062 (2)	0.060 (2)	−0.0424 (19)	−0.0181 (18)	0.0107 (17)
O3	0.063 (2)	0.055 (2)	0.059 (2)	−0.0233 (17)	−0.0031 (17)	0.0232 (17)
C1	0.033 (2)	0.042 (2)	0.026 (2)	−0.0120 (18)	−0.0035 (17)	0.0023 (17)
C2	0.033 (2)	0.040 (2)	0.034 (2)	−0.0144 (19)	−0.0044 (17)	0.0017 (18)
C3	0.039 (2)	0.051 (3)	0.032 (2)	−0.013 (2)	−0.0081 (18)	0.0002 (19)
C4	0.038 (2)	0.040 (2)	0.032 (2)	−0.0050 (19)	0.0018 (18)	0.0087 (18)
C5	0.037 (2)	0.032 (2)	0.033 (2)	−0.0111 (18)	−0.0016 (18)	0.0033 (17)
C6	0.039 (2)	0.040 (2)	0.035 (2)	−0.0142 (19)	−0.0066 (18)	0.0030 (18)
C7	0.038 (2)	0.048 (3)	0.029 (2)	−0.017 (2)	−0.0046 (18)	0.0038 (19)
C8	0.048 (3)	0.064 (3)	0.036 (2)	−0.025 (2)	−0.006 (2)	0.012 (2)
C9	0.046 (3)	0.047 (3)	0.042 (3)	−0.013 (2)	0.000 (2)	0.012 (2)
C10	0.068 (3)	0.041 (3)	0.043 (3)	−0.020 (2)	0.006 (2)	0.003 (2)
C11	0.063 (3)	0.043 (3)	0.056 (3)	−0.008 (2)	0.011 (3)	0.012 (2)
C12	0.053 (3)	0.062 (3)	0.048 (3)	−0.024 (3)	0.003 (2)	0.014 (2)
C13	0.071 (3)	0.066 (4)	0.044 (3)	−0.022 (3)	0.014 (3)	0.005 (2)
C14	0.067 (3)	0.049 (3)	0.055 (3)	−0.007 (3)	0.025 (3)	0.004 (2)

### Geometric parameters (Å, °)

**Table:**

Zn1—O1	1.948 (3)	C5—C6	1.367 (5)
Zn1—N1	2.037 (3)	C6—H6	0.9300
Zn1—I2	2.5666 (6)	C7—H7	0.9300
Zn1—I1	2.5690 (6)	C8—C9	1.508 (6)
N1—C7	1.284 (5)	C8—H8A	0.9700
N1—C8	1.473 (5)	C8—H8B	0.9700
N2—C9	1.492 (5)	C9—H9A	0.9700
N2—C10	1.496 (5)	C9—H9B	0.9700
N2—C14	1.503 (5)	C10—C11	1.509 (6)
N2—H2	0.89 (4)	C10—H10A	0.9700
N3—O3	1.220 (4)	C10—H10B	0.9700
N3—O2	1.235 (5)	C11—C12	1.518 (6)
N3—C5	1.439 (5)	C11—H11A	0.9700
O1—C2	1.285 (5)	C11—H11B	0.9700
C1—C6	1.396 (6)	C12—C13	1.502 (7)
C1—C2	1.420 (5)	C12—H12A	0.9700
C1—C7	1.452 (5)	C12—H12B	0.9700
C2—C3	1.423 (5)	C13—C14	1.503 (7)
C3—C4	1.363 (6)	C13—H13A	0.9700
C3—H3	0.9300	C13—H13B	0.9700
C4—C5	1.398 (6)	C14—H14A	0.9700
C4—H4	0.9300	C14—H14B	0.9700
			
O1—Zn1—N1	94.96 (13)	N1—C8—H8A	109.9
O1—Zn1—I2	107.27 (10)	C9—C8—H8A	109.9
N1—Zn1—I2	113.81 (10)	N1—C8—H8B	109.9
O1—Zn1—I1	121.56 (10)	C9—C8—H8B	109.9
N1—Zn1—I1	102.97 (10)	H8A—C8—H8B	108.3
I2—Zn1—I1	114.61 (2)	N2—C9—C8	112.2 (3)
C7—N1—C8	117.3 (4)	N2—C9—H9A	109.2
C7—N1—Zn1	121.3 (3)	C8—C9—H9A	109.2
C8—N1—Zn1	121.0 (3)	N2—C9—H9B	109.2
C9—N2—C10	113.3 (3)	C8—C9—H9B	109.2
C9—N2—C14	110.7 (3)	H9A—C9—H9B	107.9
C10—N2—C14	110.1 (3)	N2—C10—C11	110.8 (4)
C9—N2—H2	111 (3)	N2—C10—H10A	109.5
C10—N2—H2	105 (3)	C11—C10—H10A	109.5
C14—N2—H2	107 (3)	N2—C10—H10B	109.5
O3—N3—O2	122.8 (4)	C11—C10—H10B	109.5
O3—N3—C5	119.6 (4)	H10A—C10—H10B	108.1
O2—N3—C5	117.6 (3)	C10—C11—C12	110.9 (4)
C2—O1—Zn1	126.5 (3)	C10—C11—H11A	109.5
C6—C1—C2	119.3 (4)	C12—C11—H11A	109.5
C6—C1—C7	114.7 (4)	C10—C11—H11B	109.5
C2—C1—C7	125.8 (4)	C12—C11—H11B	109.5
O1—C2—C1	124.3 (4)	H11A—C11—H11B	108.0
O1—C2—C3	118.5 (4)	C13—C12—C11	109.5 (4)
C1—C2—C3	117.2 (4)	C13—C12—H12A	109.8
C4—C3—C2	122.4 (4)	C11—C12—H12A	109.8
C4—C3—H3	118.8	C13—C12—H12B	109.8
C2—C3—H3	118.8	C11—C12—H12B	109.8
C3—C4—C5	119.0 (4)	H12A—C12—H12B	108.2
C3—C4—H4	120.5	C12—C13—C14	111.2 (4)
C5—C4—H4	120.5	C12—C13—H13A	109.4
C6—C5—C4	120.6 (4)	C14—C13—H13A	109.4
C6—C5—N3	118.8 (4)	C12—C13—H13B	109.4
C4—C5—N3	120.5 (4)	C14—C13—H13B	109.4
C5—C6—C1	121.4 (4)	H13A—C13—H13B	108.0
C5—C6—H6	119.3	N2—C14—C13	111.4 (4)
C1—C6—H6	119.3	N2—C14—H14A	109.3
N1—C7—C1	126.9 (4)	C13—C14—H14A	109.3
N1—C7—H7	116.5	N2—C14—H14B	109.3
C1—C7—H7	116.5	C13—C14—H14B	109.3
N1—C8—C9	109.1 (4)	H14A—C14—H14B	108.0

### Hydrogen-bond geometry (Å, °)

**Table:**

*D*—H···*A*	*D*—H	H···*A*	*D*···*A*	*D*—H···*A*
N2—H2···O2^i^	0.89 (4)	2.00 (3)	2.777 (5)	144 (5)

Symmetry codes: (i) −*x*, −*y*+1, −*z*+1.
